# Data on annotated approximate bilaterally symmetric leaf-off trees based on particle flow simulation and predefined tree crown shape^☆^

**DOI:** 10.1016/j.dib.2022.107806

**Published:** 2022-01-10

**Authors:** Štefan Kohek, Niko Lukač, Damjan Strnad, Ivana Kolingerova, Borut Žalik

**Affiliations:** a Faculty of Electrical Engineering and Computer Science, University of Maribor, Maribor, Slovenia; b Faculty of Applied Sciences, University of West Bohemia, Plzen, Czech Republic

**Keywords:** Approximate symmetry, Symmetry detection, Tree synthesis, 3D tree modeling

## Abstract

Trees are natural objects, where deviations through the branches amplify geometric data for 3D representation and bring challenges to various applications dealing with 3D models, such as compression, visualization, symmetry detection, and radiative transfer simulation. This data article describes dataset of approximately symmetric 3D tree models with manually identified predominant symmetry plane in each tree model. Parameters for procedural tree synthesis were manually adjusted to produce approximately bilaterally symmetric trees which are grouped into species with distinct features. In the last step, each tree was manually annotated with approximate symmetry plane. This dataset contains geometric data of branches, manually defined parameters for tree synthesis method, point clouds, and a division plane with a score of bilateral symmetry strength. The generated trees can be used as benchmark data for verification of approximate reflectional symmetry detection methods. Additionally, generated 3D tree models can be used for other applications requiring pregenerated trees, such as compression of tree models, instancing, decimation methods, and radiative transfer simulation and modeling.

## Specifications Table

**Table:** 

Subject	Computer Graphics and Computer-Aided Design
Specific subject area	Tree modeling; Approximate symmetry detection
Type of data	3D models (OBJ files) Point cloud data (LAS files) JavaScript Object Notation files (JSON files) Images (PNG files)
How data were acquired	3D tree models were generated by the particle flow simulation method according manually defined parameters. The generated tree models were annotated manually withing the application, which is appended in the dataset. 3D tree models were converted into LAS files with application CloudCompare (http://www.cloudcompare.org/).
Data format	Raw, analyzed, filtered (generated geometric data with manually defined parameters and manually identified symmetry plane)
Parameters for data collection	3D tree models were generated regarding manually defined parameters to produce 100 diverse trees, grouped into 10 species with distinct features. The tree models were generated by the particle flow simulation method. The tree crown boundary for each tree was manually shaped into the symmetric shape. Each tree was manually annotated by visual identification of the main symmetric plane.
Description of data collection	In the first step, parameters for each tree species were defined and multiple trees of each species were generated by the particle flow simulation method. In the next step, the tree models were exported and processed with an application to manually define symmetry plane. Symmetry plane was exported into JSON file, describing parameters of each individual tree.
Data source location	Institution: Faculty of Electrical Engineering and Computer Science, University of Maribor City/Town/Region: Maribor Country: Slovenia
Data accessibility	All data [7] are supplied with this article and in the following repository: Repository name: Mendeley Direct URL to data: https://data.mendeley.com/datasets/d395bkg8ww/1 Data identification number: https://doi.org/10.17632/d395bkg8ww.1

## Value of the Data

• Comprehensive comparison between approximate symmetry detection methods requires reference benchmark data with characteristics resembling natural objects.
• Primarily, this data can benefit anyone interested in developing new approximate symmetry detection methods and performing comparison between these methods.
• The geometric data of trees and manually identified approximate symmetry planes data can be directly used as a reference data for approximate symmetry detection methods.
• Additionally, generated 3D tree models and tree skeleton can be used in any other applications, requiring pregenerated tree models, such as compression of tree models, instancing and decimation for visualization, radiative transfer modeling, simulation of forest management and horticultural practices, such as thinning and pruning.
• A set of manually identified symmetry planes and defined tree synthesis parameters, images of trees, and geometric data can provide learning datasets for new machine learning applications (e.g. for approximate symmetry detection, tree detection).

## 1 Data Description

**Fig. 1:**
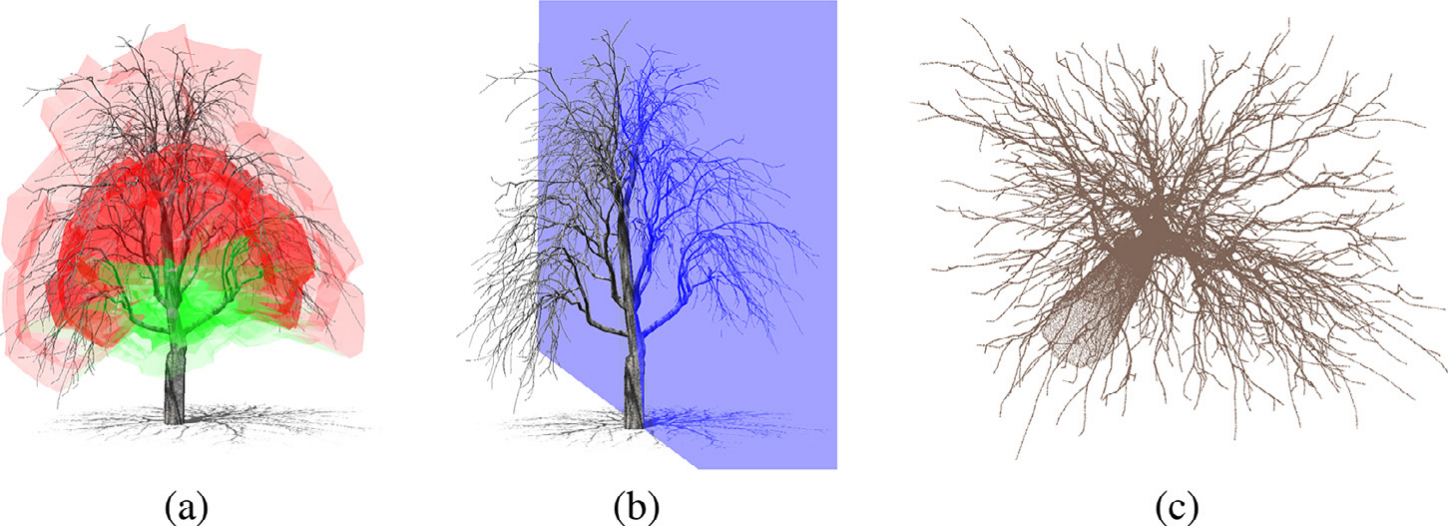
Example of one generated tree, where (a) tree crown was manually shaped into approximately symmetric form. (b) Approximate symmetry plane was manually placed into the scene. (c) Point cloud of the generated tree.

**Fig. 2:**
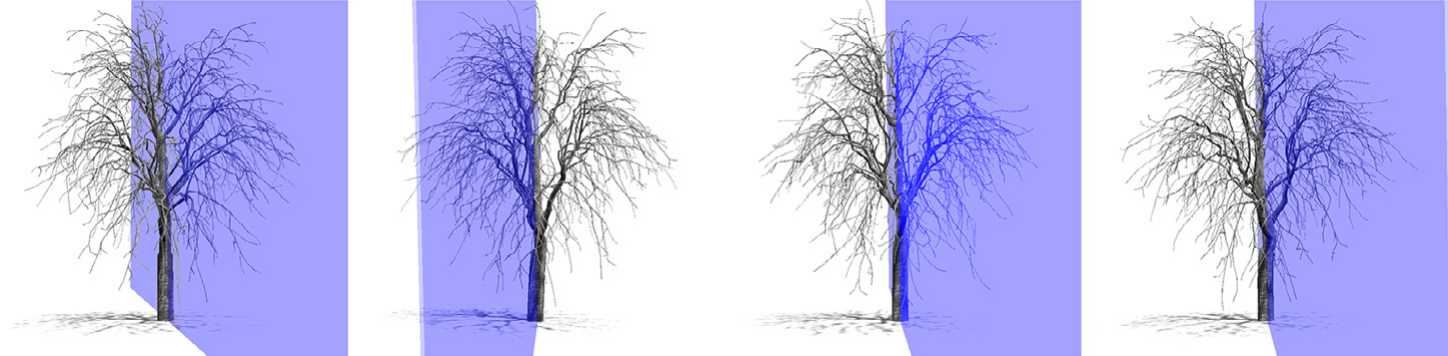
Four tree models of the same species with different symmetry planes.

**Fig. 3:**
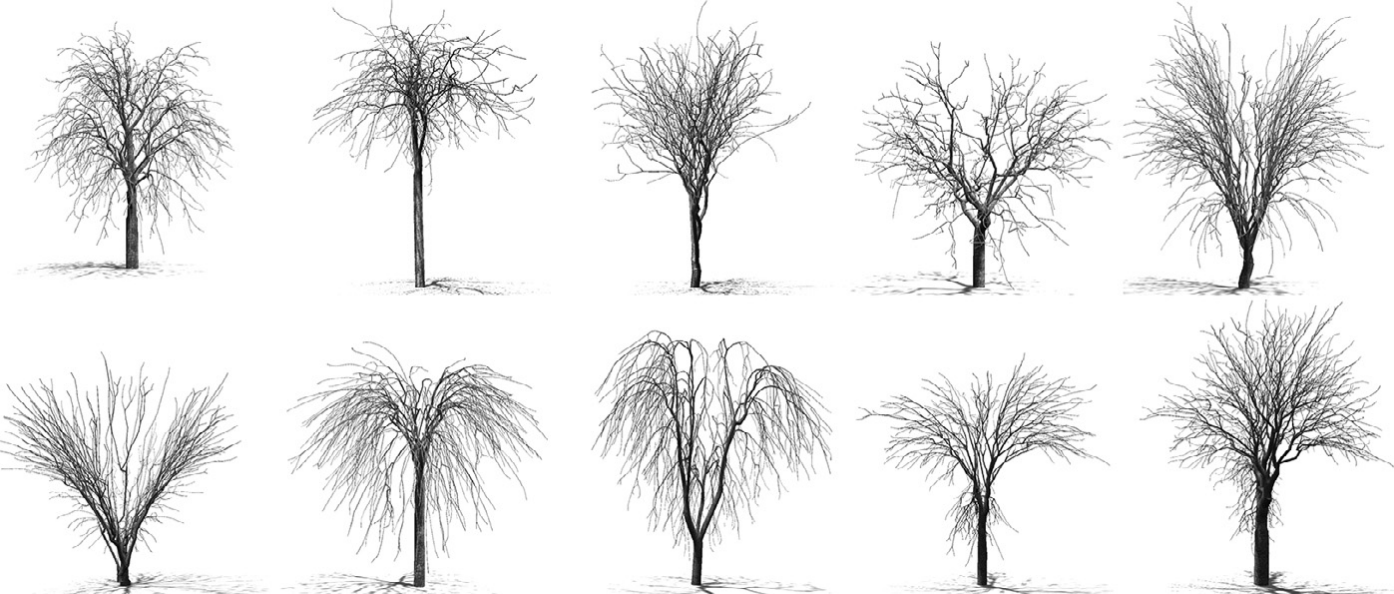
Tree examples of different species, one example for each species.

The data set provides 3D geometric data of leaf-off trees and manually specified parameters for each tree. The dataset is organized into 10 folders, which represent individual tree species. Each folder contains data of ten trees, which are numbered with the consecutive number. Detailed description of data for each tree is given in Table 1. Additionally, the dataset contains MTL file, which contains material settings for visualization of tree branches. Example of dataset for one tree is shown in Fig. 1. As shown in Fig. 1, Fig. 2, Fig. 3, trees of the same species look similar but trees of different species have distinct features.

**Table 1:** Contents of the data set for each tree.

File name	Description
*tree_[ID]_branches.obj*	Wavefront OBJ file of 3D tree models, which contains branches represented with a set of triangles. Each triangle vertex contains position, normal and texture coordinates for potential visualization.
*tree_[ID]_branches.las*	LAS file, which contains point cloud of branches.
*tree_[ID]_skeleton.obj*	Wavefront OBJ file of 3D tree skeleton, which contains branches represented with a set of lines.
*tree_[ID]_branch_tips.obj*	Wavefront OBJ file of 3D branch tips, which contains a set of vertices corresponding to branch tips. The vertices represent the tree crown shape and can be used for placement of leaves [1] or branchlets [2].
*tree_[ID].png*	Screenshot of the tree.
*tree_[ID]_plane.png*	Screenshot of the tree with included symmetry plane.
*tree_[ID]_parameters.json*	JSON file with parameters of tree synthesis method and symmetry plane. Symmetry plane is represented in a standard form of plane equation. Additionally, symmetry plane description contains value ∈[0,1] of empirically defined symmetry strength, where value 0 represents no symmetry and value 1 represents perfect symmetry.

Additionally, the dataset contains source code of the application for interactive manual approximate symmetry identification. The application is written in programming language C++ and requires library Qt 5 [3]. The application supports loading OBJ files of provided tree models and JSON files (through open file dialog or drag and drop), visualization of trees and symmetry planes, manual placement of symmetry planes, and export of symmetry planes into the JSON file.

## 2 Experimental Design, Materials and Methods

Symmetry detection in artificial objects with perfect geometric information is well explored [4], however, the problem of approximate symmetry detection in natural objects with incomplete geometry remains a challenging task [5], [6]. A comparison of new algorithms with the previous work requires good benchmark datasets and trees are typical imperfect natural phenomena and, consequently, a good example for benchmark dataset. The tree models for this data article were generated by the particle flow simulation method [1], which enables interactive direct shaping of the generated tree crown.

In the first step, the tree crown for each tree species was manually shaped into approximately symmetric shape, which is distinct mainly in one direction. This approach enables generating trees with strong primary symmetry plane. In the next step, the particle flow simulation method generated particles inside the tree crown boundary and iteratively moved generated particles towards each other and the roots. The tree skeleton was generated from trails of the moving particles. Neighboring particles were merged through the simulation, which produced the branching structure. The particle flow simulation was performed on the basis of manually predefined parameters for each species, which are listed in Table 2. The density of each tree crown was manually adjusted to create between 500 and 1000 particles. For each tree species, ten trees with different randomly placed initial particle positions were generated, which produced similar looking trees. For all trees, simulation step, Δt, was set to 0.5. Detailed description of parameters is in the original paper of the tree synthesis method [1].

**Table 2:** Parameters of species.

Species nr.	Initial attractor height - $y_{0}$	Attractor sinking speed - s	α	$w_{a}$	$w_{d}$	$w_{o}$
1	19.5	−2.8	6	1	1	1
2	20	−2	6	1	1	2
3	10	−2.6	4	1	2	1
4	9.9	−1.5	6	1	1	2
5	10	−1.9	3	2	2	1
6	4.2	−0.7	1	2	1	1
7	20	−2	1	2	1	1
8	40	−5	6	2	1	1
9	10	−1	6	2	1	1
10	10	−1	6	1	1.2	1

For each generated tree, initial positions of particles (i.e., branch tips) and the tree skeleton were collected. In the next step, generated tree skeleton was converted into geometric representation – 8-sided truncated cones. Many approximate symmetry detection methods work directly on point clouds. Therefore, mesh of tree branches was converted into the point cloud. For this task, tool CloudCompare^1^ was used. Mesh of each tree was sampled to produce $10^{6}$ points per tree.

In the last step, the generated trees were manually annotated with symmetry planes. Therefore, custom application was developed at first. The application enables 3D visualization of trees and manual placement of symmetry plane. By using this application, the most noticeable symmetry plane was visually empirically identified for each tree. In addition, each symmetry plane was augmented with a value, where 0 resembles no symmetry and 1 resembles perfect symmetry.

## CRediT authorship contribution statement

**Štefan Kohek:** Conceptualization, Writing – original draft, Methodology, Software, Data curation. **Niko Lukač:** Conceptualization, Writing – review & editing, Methodology, Project administration. **Damjan Strnad:** Conceptualization, Writing – review & editing, Methodology. **Ivana Kolingerova:** Conceptualization, Writing – review & editing, Funding acquisition. **Borut Žalik:** Conceptualization, Writing – review & editing, Funding acquisition, Supervision.

## Declaration of Competing Interest

The authors declare that they have no known competing financial interests or personal relationships which have, or could be perceived to have, influenced the work reported in this article.

## Footnotes

1 http://www.cloudcompare.org/
